# Crosstalk between Extracellular Vesicles and the Tumor Microenvironment: Mechanistic Insights and Therapeutic Opportunities

**DOI:** 10.32604/or.2026.079562

**Published:** 2026-07-16

**Authors:** Yaqi Xu, Jia Zhao, Xiaowen Mao

**Affiliations:** 1 State Key Laboratory of Mechanism and Quality of Chinese Medicine, Institute of Chinese Medical Sciences, University of Macau, Macao SAR, China; 2 School of Chinese Medicine, Li Ka Shing Faculty of Medicine, The University of Hong Kong, Hong Kong SAR, China

**Keywords:** Extracellular vesicles, tumor microenvironment, intercellular crosstalk, immunotherapy, tumor vaccine

## Abstract

Extracellular vesicles (EVs) are actively secreted, membrane-enclosed nanoparticles that serve as pivotal mediators of intercellular communication. They function as key mediators of intercellular communication by transporting diverse biomolecules, including proteins, nucleic acids, and metabolites. Within the tumor microenvironment, EVs drive complex cellular crosstalk and critically regulate tumor progression by remodeling the extracellular matrix, conferring drug resistance, and reprogramming immune responses. Given their natural biocompatibility, tissue tropism, and ability to cross biological barriers, EVs have emerged as promising platforms for immunotherapy, tumor vaccines and targeted drug delivery system. Moreover, the rapid expansion of EV-based clinical trials highlights their promise in precision medicine. Concurrently, the limitations associated with EV-based therapeutic strategies are critically evaluated to inform future development. This review has also detailed the importance of single-vesicle analysis, which represents a rapidly evolving frontier in EV science. Aiming to bridge the gap between mechanistic understanding and clinical practice, this review delineates the pivotal roles of EV-mediated communication in reshaping the dynamic homeostasis of the tumor microenvironment. Moreover, we provide a critical analysis of current EV-based therapeutic pipelines and their translational challenges, offering key perspectives and theoretical support for the future of precision medicine.

## 1 Introduction

Intercellular signaling mechanisms are essential for homeostatic maintenance and facilitate rapid cellular adaptation to dynamic environmental changes. Classically, this communication is mediated through direct cell-to-cell contact and the exchange of bioactive molecules [1]. Recent studies have demonstrated that extracellular vesicles (EVs) act as pivotal mediators of intercellular communication, enabling both short- and long-range cellular communication [2].

EVs are lipid bilayer-enclosed nanoparticles released by cells that lack self-replication capacity. Historically, EVs have been named according to various criteria such as size, biogenetic pathway, and cellular origin. In line with the 2023 Minimal Information for Studies of Extracellular Vesicles (MISEV) recommendations, EVs can be broadly categorized by diameter into small EVs (<200 nm) and large EVs (>200 nm). Nevertheless, in biological research, classification based on EVs biogenesis remains more prevalent. Exosomes, typically under 200 nm, derive from the endosomal system [3]. Ectosomes (including microvesicles and microparticles) are generated by direct outward budding of the plasma membrane and exhibit a wider size distribution [3]. Emerging research has identified additional EV populations, including migrasomes [4] and blebbisomes [5], further expanding EV classification.

Common protein markers used to characterize EVs include transmembrane proteins such as tetraspanins proteins (cluster of differentiation 9 [CD9], CD63, CD81) and flotillin-1/2, as well as biomarkers associated with EV biogenesis, including Tumor susceptibility gene 101 (TSG101), apoptosis-linked gene 2-interacting protein X (ALIX), annexin A1, and solute carrier family 3 member 2 (SLC3A2) [3,6,7]. It should be noted that no single biomarker currently exists that adequately captures the heterogeneity of the EV population or can reliably distinguish between EV subtypes.

Under normal physiological conditions, EVs function as key mediators of intercellular communication, facilitating the exchange of bioactive proteins, lipids, and nucleic acids between donor and recipient cells [8]. The composition of EV cargo is dynamic and influenced by the type and state of the donor cells, as well as by changes in the external environment [9]. Once released, EVs can travel via bodily fluids over short or long distances and are taken up by recipient cells through mechanisms such as membrane fusion, receptor-mediated uptake, or endocytosis, thereby enabling the transfer of EV cargo and modulation of recipient cell function [10].

**Figure 1:**
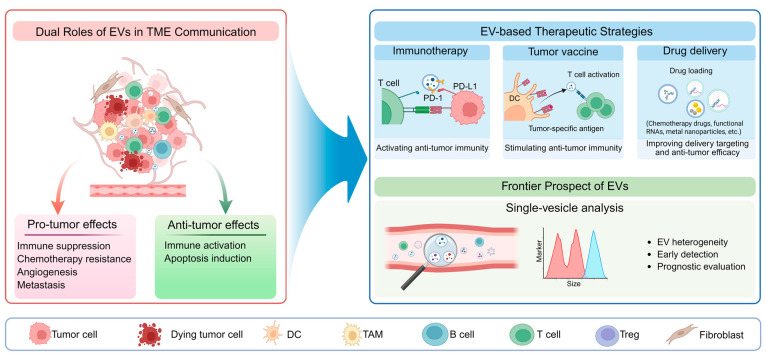
Current Landscape and Future Frontiers of Extracellular Vesicles-Based Therapies in Cancer. Extracellular vesicles (EVs) mediate complex intercellular communication within the tumor microenvironment (TME) and exert binary effects on the tumor progression. On the one hand, EVs promote tumor progression through immunosuppression, chemoresistance, angiogenesis, and metastasis. On the other hand, they also manifest anti-tumor effects via immune activation and the induction of apoptosis. Based on these biological mechanisms, diverse novel therapeutic approaches are being explored, categorizable into immunotherapy, which modulates the TME immune response and stimulates anti-tumor immunity; tumor vaccines, that activate anti-tumor immunity by delivering tumor-specific antigens; and drug delivery, leveraging EVs for cargo loading (e.g., small molecules, siRNA) and targeted delivery to tumor cells. Furthermore, advancements in single-EV analysis techniques facilitate the dissection of EV heterogeneity and hold immense potential for applications in early tumor diagnosis and prognosis assessment. DC: Dendritic Cell; TAM: Tumor-Associated Macrophage.

The hypermetabolic state of malignant cells and perturbations of the tumor microenvironment (TME) drive abundant EV release into circulation [11,12]. Tumor cell-derived EVs actively remodel the extracellular matrix (ECM) to establish an environment suitable for tumor cell survival and proliferation [13]. Additionally, EVs within the TME facilitate tumor progression by potentiating tumor angiogenesis [14], participating in pre-metastatic niche formation [15], mediating immune evasion [16] and conferring chemoresistance via transfer of EV cargo [17]. Current efforts are increasingly focused on unraveling the complex interactions between EVs and the TME, alongside the development of novel therapeutic strategies aimed at disrupting EV-mediated intercellular crosstalk in cancer progression. Our review synthesizes emerging evidence on the dynamic interplay between EVs and the TME, elucidating key molecular mechanisms underlying their pro-tumorigenic interactions. Furthermore, we discuss the emerging therapeutic strategies designed to disrupt EV-TME communication for clinical benefit (Fig. 1).

## 2 EV-Driven Crosstalk among TME Cells

TME represents a complex, heterogeneous ecosystem characterized by non-cellular structural elements and a sophisticated cellular landscape, including phenotypically distinct tumor cells, immune cells (e.g., dendritic cells, lymphocytes, and macrophages), stromal cells, endothelial cells, and cancer-associated fibroblasts (CAFs) [18]. These cellular constituents actively secrete EVs that mediate intercellular crosstalk, collectively shaping the dynamic TME landscape and driving tumor progression [19].

### 2.1 Effects of EVs in Tumor Cell

Accumulating evidence identifies EVs as critical mediators of tumor progression and metastatic dissemination. Specifically, EVs participate in multiple steps of the metastatic cascade, either by directly conferring invasive properties to cancer cells or by delivering matrix metalloproteinases (MMPs) that degrade the ECM, thereby facilitating tumor cell dissemination [20,21]. Zhang et al. reported that therapy-induced senescence in colorectal cancer cells drives tumor progression through senescence-associated secretory phenotype-mediated EV secretion. Specifically, senescent cells produce serpin family E member 1-enriched EVs that, following recipient cell uptake, orchestrate NF-κB activation via p65 binding and nuclear translocation, establishing a pro-tumorigenic feedback loop [22].

MicroRNA (miRNA) represent a primary class of functional cargoes encapsulated within EVs, serving as pivotal regulators of post-transcriptional gene expression upon uptake by recipient cells [23]. The study by Li and colleagues revealed that EV-mediated intercellular communication from M2 tumor-associated macrophages (TAMs) to Non-small cell lung cancer (NSCLC) cells involves selective enrichment of oncogenic miRNAs (miR-155/miR-196a-5p), which significantly enhance metastatic potential both *in vitro* and *in vivo* [24].

EVs have been established as critical mediators of therapeutic resistance in cancer [25]. EVs contribute to chemotherapy resistance via several key mechanisms. These include the horizontal transfer of functional cargo, such as drug efflux pumps and anti-apoptotic proteins, into recipient cells. This process reduces intracellular drug accumulation and attenuates apoptosis, thereby promoting tumor cell survival under therapeutic pressure. Chemotherapy-induced EVs (chemo-EVs) undergo molecular remodeling, carrying elevated levels of pro-tumorigenic factors that critically influence tumor behavior [26]. Chemotherapeutic agents further enhance EV secretion from tumor cells [27]. Specifically, Kreger et al. demonstrated that that paclitaxel treatment triggers the release of survivin-enriched EVs from breast cancer cells, which promotes cancer cell survival and confers chemoresistance to recipient cells [28].

Furthermore, EVs induce transcriptional reprogramming within tumor cells by delivering oncogenic miRNAs and lncRNAs, activating pro-survival signaling pathways and leading to chemotherapy resistance. The immunosuppressive TAM-EV miRNA cargo plays a pivotal role in therapeutic resistance. Specifically, Binenbaum et al. demonstrated that pancreatic ductal adenocarcinoma (PDAC)-bearing mice treated with macrophage-derived EVs developed resistance to gemcitabine, the first-line therapeutic agent for PDAC. This resistance was mediated by TAM-secreted miR-365, which impairs gemcitabine activity via two distinct pathways. Mechanistically, miR-365 involves the expansion of the triphospho-nucleotide pool, which exerts competitive inhibition on gemcitabine, alongside the induction of cytidine deaminase-mediated drug inactivation [29].

In summary, EVs mediate the transfer of diverse oncogenic cargo within the TME, driving metastatic dissemination and chemoresistance in recipient cancer cells. Therefore, targeting EV-mediated intercellular communication networks may offer a theoretical framework for developing novel strategies to suppress tumor progression and overcome clinical resistance.

### 2.2 Crosstalk of EVs and Endothelial Cells in the TME

EVs transport diverse protein and nucleic acid cargoes that actively regulate endothelial cells within the TME, promoting angiogenesis within tumors, reducing cell permeability, and facilitating pre-metastatic niche formation [30]. Specifically, EVs from senescent fibroblasts promote tumor angiogenesis and endothelial sprouting through a unique molecular signature marked by CD9 downregulation and ANGPTL2 enrichment [31]. Similarly, gastric cancer-derived EVs (GC-EVs) deliver angiopoietin-2 (ANG2) to activate the Phosphoinositide 3-kinase/protein kinase B (PI3K/AKT) pathway in endothelial cells, thereby stimulating angiogenesis [32]. In metastatic breast cancer, EV-encapsulated miR-105 disrupts endothelial barrier integrity by downregulating tight junction proteins, facilitating hematogenous dissemination. Furthermore, tumor EV-derived non-coding RNAs, including miR-92a-3p [33], miR-183-5p [34], and the long non-coding RNA UBE2CP3 [35], mediate intercellular crosstalk between tumor cells and endothelial cells. S100A16 encapsulated in the endothelial cell-derived EVs (EC-EVs) promotes resistance to apoptosis and facilitates brain tissue colonization by small cell lung cancer cells [36]. These examples underscore the cargo-dependent nature of EC-EV mediated intercellular communication in cancer progression.

Elucidating EV-endothelial cell interactions may help enhance the efficacy of anti-angiogenic drugs and establish a theoretical basis for the development of highly effective targeted drugs.

### 2.3 Dual Regulation of EVs on Immune Cells in the TME

Tumor-associated macrophages (TAMs) constitute a pivotal cellular component in the TME and are often thought to be associated with poor prognosis [37]. For decades, macrophages have been conventionally classified into two main phenotypes: the pro-inflammatory M1 type and the immunosuppressive M2 type. M1 exert anti-tumor effects and secret pro-inflammatory cytokine, whereas M2 promote tumor progression [38]. The relative proportion of M1 and M2 in the TME serves as a determinant of tumor progression, with a lower M1/M2 ratio typically associated with advanced disease stages and therapy resistance. However, this M1/M2 classification is now considered oversimplified, as it ignores the dynamic heterogeneity of TAMs and the complexity of the TME [39]. Enabled by the rapid advancement of single-cell technologies, TAMs are now subdivided into more refined subpopulations based on distinct functional states and gene signatures. These include interferon-primed TAMs, immune regulatory TAMs, inflammatory cytokine-enriched TAMs, lipid-associated TAMs, pro-angiogenic TAMs, tissue-resident macrophage-like TAMs, and glycolytic TAMs [37,40].

Likewise, other immune cell populations play critical roles within the TME. As innate immune effectors, natural killer (NK) cells eliminate tumor cells through the release of perforin and granzymes upon activation, constituting an important component of cancer immunotherapy [41]. Dendritic cells (DCs), as professional antigen-presenting cells, recognize and process tumor-associated antigens to initiate adaptive immune responses [42]. Among adaptive immune cells, T cells are central to antitumor immunity: helper T cells and cytotoxic T cells respectively coordinate immune responses and directly kill tumor cells, whereas regulatory T cells (Tregs) suppress immune activity and contribute to tumor immune evasion [43].

EVs serve as pivotal mediators of communication between immune cells—such as macrophages, NK cells, T cells, and B cells—and tumor cells in the TME. Through the transfer of immunosuppressive or immunostimulatory molecules, EVs dynamically reprogram the local immune microenvironment.

#### 2.3.1 Immune Activation

During tumor initiation, tumor-derived extracellular vesicles (TDEVs) contribute to the formation of a microenvironment centered around M1 macrophages [30]. TDEVs contain cargoes, including miR-3342 [44] and miR-125b-243 [45], which can exert anti-tumor effects by directly polarizing macrophages to M1 or reprogramming M2 macrophages to M1. DC-derived EVs carrying TNF-α activate natural killer (NK) cells via TNF receptor binding, stimulating interferon-γ (IFN-γ) production [46].

TDEVs mediate the activation of T cells and NK cells, driving a powerful anti-tumor effect. TDEVs loaded with the miRNAs Let-7i, miR-142, and miR-155 potentiate cytotoxic T cell activity, consequently inhibiting tumor progression in a murine model [47]. Moreover, TDEVs can enhance the antigen-presenting capacity of DCs, thereby improving anti-tumor efficacy. Specifically, EVs released from genetically engineered tumor cells exhibit high immunogenicity, which promotes DC maturation and further augments anti-tumor activity [48].

In summary, EVs contribute to immune activation in the TME by promoting M1 macrophage polarization, activating anti-tumor immune effectors, and facilitating antigen presentation, resulting in a potent anti-tumor outcome.

#### 2.3.2 Immune Suppression

However, as tumors progress and the TME evolves, EVs contribute to the formation of an immunosuppressive microenvironment dominated by M2 [30]. For example, tissue inhibitor of metalloproteinase 3 (TIMP3) is a well-established tumor suppressor. In hepatocellular carcinoma (HCC), Hu et al. demonstrated that TDEVs facilitate M2 macrophage polarization by delivering miR-452-5p to downregulate TIMP3 expression, thereby accelerating malignant progression through the establishment of an immunosuppressive niche [49].

EVs from various cellular origins function as potent immunosuppressive agents, not only directly inhibiting T cell activity but also promoting their exhaustion, thereby dampening the overall anti-tumor immune response. M2 macrophage-derived EVs deliver miR-29a-3p and miR-21-5p to CD4^+^ T cells, activating signal transducer and activator of transcription 3 signaling and shifting the Treg/Th17 balance toward immunosuppression [50,51]. Ramil et al. found that immunosuppressive myeloid-derived suppressor cells (MDSCs) educated by CAFs release EVs containing fructose bisphosphatase 1, directly inhibiting T cell function [52]. The release of Treg-derived EVs (Treg-EVs) further reinforce the immunosuppressive microenvironment by polarizing macrophages toward an M2 phenotype, promoting the formation of tolerogenic DCs, and inhibiting the proliferation and activation of effector T cells [53].

The effects of TDEVs on immune cells are not a binary opposition of activation and suppression but are instead highly context-dependent. For instance, EVs engineered from myeloid leukemia cells to express the immunostimulatory ligands IL-15, IL-18, and 4-1BBL initially activate and enhance the proliferation of NK cells, thereby promoting anti-tumor activity. However, prolonged exposure for 48 h paradoxically suppresses the cytotoxic function of these NK cells [54].

The TME harbors EVs that display an immunomodulatory duality, capable of both activating anti-tumor immunity and suppressing immune cells. This duality offers a strategic avenue for developing next-generation immunotherapies aimed at amplifying the beneficial signals and blocking the inhibitory ones carried by EVs.

### 2.4 Crosstalk of EVs and Stromal Cells in the TME

CAFs represent the most abundant stromal population within the TME, playing an important role in angiogenesis, tumor growth and metastasis, and maintaining an immunosuppressive microenvironment by engaging in extensive intercellular crosstalk within the TME [55]. In cancer, CAFs can be transformed from normal fibroblasts [56], mesenchymal stem cells [57], adipocytes [58], and other cells.

EVs in the TME, particularly TDEVs, exhibit multifaceted effects on CAFs. Emerging studies have reported that TDEVs can induce the activation of lung fibroblasts to facilitate metastatic progression. Our team reported that Nidogen 1 in EVs derived from metastatic liver cancer cells is associated with the activation of lung fibroblasts, thereby enhancing tumor cell growth and invasion and pulmonary colonization [15]. Coincidentally, CAF-derived EVs have also demonstrated similar results. In salivary adenoid cystic carcinoma, Kong et al. found that CAF-derived EVs carrying ITGα2β1 facilitate EV uptake by lung fibroblasts and subsequent niche formation [59]. These findings collectively highlight integrin-mediated EV targeting as a promising therapeutic avenue for metastatic intervention.

Given the striking heterogeneity of CAF populations, TDEVs serve as key effectors that drive the functional diversification of fibroblasts within the TME. For instance, breast cancer-derived exosomes containing survivin upregulate superoxide dismutase 1 (SOD1) in resident fibroblasts, promoting their conversion into tumor-promoting myofibroblasts that secrete pro-tumorigenic factors to enhance cancer proliferation and metastatic dissemination [56]. Furthermore, EV-mediated crosstalk between tumor cells and fibroblasts facilitates the emergence of specialized CAF subtypes, including inflammatory CAFs (iCAFs) [55,60] and vascular CAFs (vCAFs) [61], which contribute to tumor progression through distinct mechanisms.

Similarly, CAF-derived EVs influence tumor cell behavior by enhancing malignant progression, metastatic potential, and therapy resistance through multifaceted mechanisms.

### 2.5 Anti-Tumor Functions of EVs in the TME

While EVs are widely found to promote tumor progression, a growing body of evidence from both *in vitro* and *in vivo* studies demonstrates that EVs can also exert potent anti-tumor responses by inhibiting proliferation and inducing apoptosis in cancer cells.

For instance, EC-EVs deliver miR-503 to tumor cells, markedly suppressing their proliferative, migratory, and invasive capacities [62]. Studies employing a simulated TME model have elucidated that the apoptotic effect in pancreatic cancer cells is mediated by miR-145 delivered via EVs originating from tumor-associated stromal cells [63]. EVs secreted by NK cells carry anti-tumor miRNAs. These EVs can trigger caspase-mediated apoptosis and suppress proliferation in tumor cells [64].

**Figure 2:**
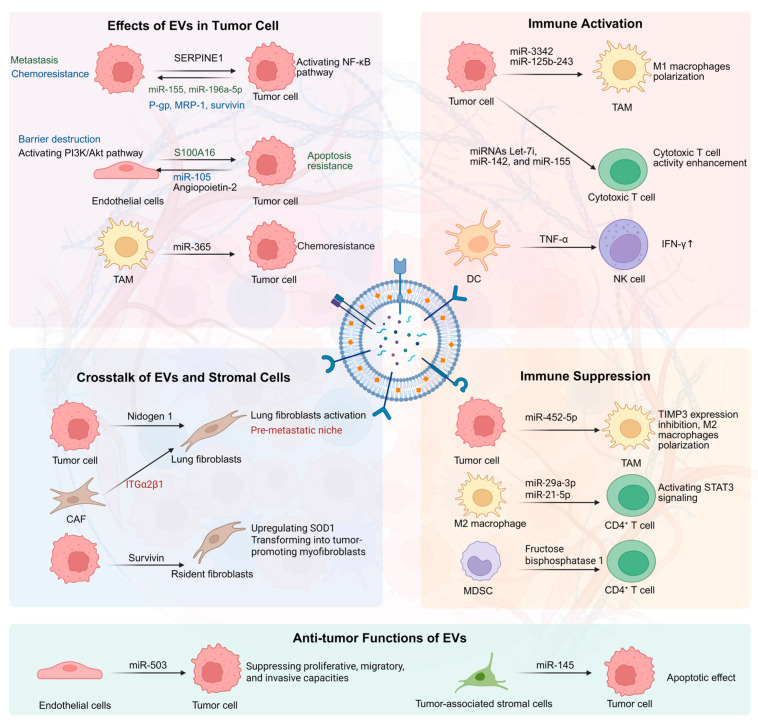
Extracellular vesicles (EVs) mediate intercellular communication within the tumor microenvironment (TME). EVs, released by diverse cell types within the TME, serve as crucial mediators of intercellular communication. This EV-driven crosstalk contributes to tumor progression by facilitating apoptosis resistance, angiogenesis, drug resistance, and immunosuppression. Conversely, EVs can also exert tumor-suppressive effects through the activation of antitumor immunity and the induction of tumor cell apoptosis. TAM: Tumor-Associated Macrophage; DC: Dendritic Cell; CAF: Cancer-Associated Fibroblast.

Nevertheless, research on the anti-tumor effects of EVs remains limited. Most studies are confined to *in vitro* models of the TME, which fail to fully recapitulate the complexity of *in vivo* conditions. Advancing this research direction is crucial for harnessing the therapeutic potential of EVs, underscoring the need for more intensive research efforts in this field (Fig. 2).

EVs facilitate tumor metastasis via three core mechanistic pathways. First, they directly transfer functional proteins, such as P-glycoprotein (P-gp) [65] and survivin [28], to recipient cells, conferring immediate phenotypic changes including chemotherapy resistance and enhanced cell survival. Second, EVs deliver regulatory molecules like non-coding RNAs (e.g., miR-155) [24] and cytokines (e.g., TNF-α) [46] to recipient cells, where they induce transcriptional reprogramming and modulate long-term cellular behaviors such as metastatic capacity and EMT. Third, EVs carry functional proteins that act on endothelial cells and fibroblasts, promoting angiogenesis, disrupting endothelial barrier integrity, and fostering the formation of a pre-metastatic microenvironment, all of which collectively facilitate tumor dissemination. These EV-mediated effects converge on key signaling pathways, notably PI3K/Akt [34] and NF-κB [22], forming an integrated regulatory network that drives tumor progression.

## 3 Effects of the TME on EVs

Beyond their role in mediating intercellular communication, EV biogenesis and functionality are profoundly influenced by the dynamic characteristics of TME. This section will systematically review how key TME features influence EV production, cargo sorting, and subsequent biological effects.

### 3.1 TME with Hypoxia Characteristics

Hypoxia constitutes a hallmark of solid tumors, driven by a profound imbalance between accelerated cell proliferation and inadequate vascular oxygen delivery, forcing tumor cells to undergo metabolic reprogramming. Hypoxia is involved in various processes related to cancer progression and remains a major driver of tumor resistance and recurrence [66].

In response to the hypoxic TME, cells within the TME secrete more EVs to meet intercellular communication demands, thereby influencing tumor progression. However, the underlying mechanisms remain incompletely understood. While the balance between multivesicular bodies (MVB) degradation and secretion is a key yet underexplored regulator of EV release, hypoxia is known to tip this balance toward secretion [67]. Specifically, it promotes the intracellular trafficking and plasma membrane fusion of MVBs, thereby elevating EV secretion. HOX transcript antisense RNA (HOTAIR) is a long non-coding RNA (lncRNA) frequently upregulated in various cancers. Research by Yang et al. revealed that HOTAIR upregulates the expression of RAB35, a key regulator of intracellular MVB transport, thereby enhancing MVB trafficking to the plasma membrane. Beyond this, HOTAIR overexpression also promotes the co-localization of vesicle-associated membrane protein 3 (VAMP3) with SNAP23 on MVB membranes, a step that facilitates subsequent MVB-plasma membrane fusion. Finally, phosphorylation of SNAP23 acts as the terminal trigger in this cascade, directly driving the secretion of EVs [68]. Another potential mechanism is that hypoxia-mediated disruption of intracellular MVB trafficking blocks the lysosomal fusion and degradation pathway. This redirection of MVB fate consequently promotes increased EV secretion. For example, in hypoxic head and neck squamous cell carcinoma (HNSCC), HIF-1α-mediated transcriptional repression reduces the expression of ATP6V1A, an essential V-ATPase subunit required for lysosomal homeostasis. The resulting lysosomal dysfunction disrupts MVB-lysosome fusion, thus promoting elevated EV secretion [69].

Hypoxia profoundly remodels the molecular cargo of EVs to exert pro-tumorigenic effects on recipient cells. EVs derived from mesenchymal stem cells under hypoxic conditions are enriched with miR-21-5p, which promotes tumor cell proliferation, metastatic dissemination, and M2 macrophage polarization [70]. EVs secreted by hypoxia-polarized M2-like TAMs deliver circ_0003137 to induce EMT in glioblastoma cells, thereby driving tumor progression and metastasis [71]. In addition, TDEVs from hypoxic TME carry pro-angiogenic miRNAs, including miR-135b [72] and miR-23a [73], which stabilize HIF-1α in endothelial cells through multiple mechanisms, ultimately promoting tumor angiogenesis. Additionally, hypoxia within the TME promotes the polarization of TAMs to an M2 phenotype. These M2-type TAMs release EVs enriched with cytokines and growth factors, which activates endothelial cells, promoting angiogenesis [74,75].

While the hypoxic TME enhances tumor cell uptake of EVs [76], how hypoxia alters extracellular EVs transport remains unexplored. Taken together, hypoxia governs a sophisticated web of EV-based crosstalk in the TME, which facilitates tumor adaptation and remodeling. Thus, interfering with hypoxic signaling or its downstream pathways offers a viable approach for developing novel anti-tumor therapies that target EV biology.

### 3.2 TME with Acidic Characteristics

Metabolic reprogramming drives tumor cells to secrete excessive lactate, whose accumulation establishes an acidic TME [77]. Recent studies have demonstrated that acidic TME orchestrates multifaceted pro-tumor effects, including suppression of immune cell function [78], evasion of immune surveillance [79], stimulation of angiogenesis [80], and induction of therapy resistance [81]. Collectively, these processes culminate in tumor invasion and metastasis.

Intercellular communication via EVs is profoundly sensitive to TME acidification, which alters vesicle biogenesis, transport, and uptake kinetics. First, an acidic microenvironment drives increased biogenesis, cargo reprogramming and secretion of EVs. In melanoma, Boussadia et al. demonstrated that in acidic TME, TDEVs are enriched with pro-invasive proteins, which confer heightened metastatic potential to tumor cells under neutral pH conditions [82]. Furthermore, an *in vitro* study indicates that acidic culture medium increases the yield of both proteins and nucleic acids in EVs. In contrast, alkaline conditions, or the use of proton pump inhibitors, suppress the secretion of EVs and reduce their molecular cargo [83]. However, the mechanism by which an acidic TME promotes EV biogenesis remains unclear. One hypothesis suggests that acidic TME accelerates the cellular turnover of lysosomes and EVs, thereby increasing the rates of exosome synthesis and release [84].

Acidic conditions within the TME promote EV uptake by modulating membrane composition and properties to facilitate efficient membrane fusion between EVs and recipient cells. From a biophysical perspective, the acidic conditions reduce charge shielding on the EV membrane, thereby decreasing electrostatic repulsion during fusion. Simultaneously, the stiffness of the EV membrane may become more comparable to that of the plasma membrane, further promoting fusion [85]. Moreover, changes in membrane lipid and protein composition are critical for efficient fusion between EVs and the cellular membrane. In a melanoma study, Parolini et al. demonstrated that an acidic environment not only enhances EV secretion and uptake but also alters EV membrane properties, resulting in high rigidity and elevated sphingomyelin/ganglioside GM3 content, features that may account for the improved fusion efficiency [86].

Targeting or reversing the tumor-promoting effects of the acidic TME represents a promising therapeutic strategy. The acidic TME fosters immune evasion by upregulating IFN-γ-dependent PD-L1 expression on tumor cells through STAT1/eIF4F translational activation. Neutralization of extracellular acidity has been shown to reduce PD-L1 expression while enhancing immune cell infiltration, thereby restoring antitumor immunity [87]. Building on the finding that acidic conditions promote EV uptake, a follow-up study reports the development of a smart drug delivery system specifically designed to leverage this phenomenon, which has substantially enhanced the effectiveness of tumor treatment [88]. Consequently, therapeutic interventions that either normalize the acidic microenvironment or target acid-regulated EV functions may offer a viable pathway to enhance treatment efficacy.

### 3.3 Other Features of the TME

Therapeutic pressures, including chemotherapy and radiotherapy, drive tumor cells to dynamically remodel their EV secretion, thereby modulating intercellular communication within the stressed TME. This process reduces intracellular levels of toxic byproducts or drugs, while enabling the export of oncogenic molecules, thereby contributing to acquired drug resistance. For instance, both photodynamic therapy and chemotherapies trigger the rapid release of large quantities of EVs loaded with drugs, oncoproteins, and nucleic acids. This not only diminishes treatment efficacy but may also disseminate harmful cargo to distant healthy cells, exacerbating off-target effects [89]. Under cisplatin pressure, tumor cells package the drug into EVs for secretion, a direct efflux mechanism that lowers intracellular accumulation and promotes chemoresistance [89]. Similarly, irradiated cells secrete EVs carrying specific miRNAs that mediate bystander effects, facilitating communications between irradiated and non-irradiated cells [90]. Collectively, these findings highlight how therapy-stressed tumors exploit EV secretion to alter both local and systemic cell communication, supporting survival and compromising treatment outcomes.

In the TME, remodeling of the ECM and its mechanical properties influence cancer progression, a process that in turn regulates the biogenesis, secretion, and release of EVs from cancer cells [91]. For example, a stiffened ECM can enhance EV secretion by activating the Akt signaling pathway, which in turn upregulates Rab8 activity [92]. Nutrient deprivation imposes considerable stress on tumor cells, leading to increased EV secretion and altered cargo composition [93]. However, research in this area remains limited, and the specific mechanisms by which the ECM regulates EV biogenesis and extracellular transport are still not well understood. Further investigation into these processes is needed to clarify their role in tumor progression and therapy resistance.

Thus, the defining characteristics of the TME—hypoxia, acidity, therapeutic pressure, and ECM remodeling—converge to modulate EV biogenesis and function, profoundly influencing oncogenesis and tumor progression.

## 4 Therapeutic Potential and Frontier Prospect in Cancer

As discussed previously, EVs exhibit a dual role in the TME, capable of both promoting tumorigenesis and progression, and suppressing tumor advancement and metastasis. However, growing research attention has been focused on the intricate crosstalk mediated by EVs between tumor cells and immune cells. TDEVs can modulate the crosstalk among various immune cells within the TME to facilitate cancer growth and metastasis [94,95]. These mechanisms include impairing the differentiation and maturation of dendritic cells, suppressing the cytotoxicity of NK cells, and upregulating the population of Tregs, among others [96]. In-depth research into the mechanisms by which EVs participate in tumor immune responses will provide a more robust foundation for developing and applying effective strategies for cancer immunotherapy.

### 4.1 Potential of EVs in Cancer Treatment

#### 4.1.1 Immunotherapy

The immunosuppressive tumor microenvironment represents a critical driver of malignant progression. To restore a normal immune environment, immunotherapy aims to restore and enhance immune system function, thereby killing tumor cells without harming the body. However, clinical trial results indicate that immunotherapy yields clinical responses in only a subset of patients, while the majority of patients still experience limited therapeutic benefit due to immune escape and tumor heterogeneity [97].

As mediators of intercellular crosstalk within the TME, EVs hold promise as tools for the development of immunotherapeutic strategies [98]. EVs’ inherent tumor targeting, biocompatibility, and barrier-crossing abilities have led to their initial application in immunotherapy. Given their ability to carry functional cargo, EV modification could allow them to deliver target molecules to recipient cells and exert their functions.

While immune checkpoint inhibitors (ICIs) have revolutionized cancer immunotherapy, their efficacy remains limited by the immunosuppressive TME [99]. Engineered EVs offer a promising alternative for immune checkpoint blockade, capable of restoring anti-tumor immunity with improved safety profiles. PD-1/PD-L1 axis is the star immune checkpoints in immunotherapy, as its interaction enables tumor cells to evade T cell-mediated immunity [100]. Although monoclonal antibodies against PD-1/PD-L1 axis can disrupt this interaction, they are associated with significant adverse effects and rapid clearance [101,102]. In recent years, many scientists have attempted to enable EVs expressing PD-1 from T cells, macrophages, and tumor cells, which can induce tumor cell apoptosis and block PD-L1-induced immune escape [103,104]. Furthermore, combination strategies employing EV-based ICIs with chemotherapy exhibit enhanced therapeutic effects [105]. This therapeutic strategy can also be applied to the interaction between CD47 on the surface of tumor cells and SIRPα on the surface of macrophages [106]. The inherent characteristics of engineered EVs make it safer and have fewer side effects in the field of blocking immune checkpoints, and they have broad prospects in the promotion and application of ICI therapy.

#### 4.1.2 Tumor Vaccines

Tumor vaccines represent a promising immunotherapeutic strategy designed to activate robust immune responses and achieve anti-tumor efficacy through delivery of tumor-specific antigens and adjuvants [107]. Owing to their superior capacity for antigen presentation, DCs have been extensively utilized in the design of tumor vaccines to prime robust T-cell-mediated anti-tumor responses. EVs secreted by DCs carry MHC peptide complexes, co-stimulatory molecules, and adhesion molecules, and possess the same antigen-presenting capacity as DCs [108]. Compared to whole-cell vaccines, EV-based vaccines exhibit superior resistance to immunosuppressive signals in the TME, making them particularly effective for T cell activation. Lu et al. found that DC-derived EVs carrying alpha-fetoprotein activated CD8^+^ cytotoxic T lymphocytes in HCC mouse model, inducing a potent anti-tumor immune response and significantly inhibiting tumor progression [109]. Combining DC-derived EVs with ICIs further amplifies the therapeutic efficacy of DC-derived EVs, representing a promising combinatorial immunotherapeutic approach [110].

Related clinical trials are ongoing. In a metastatic melanoma trial, autologous DC-generated EVs loaded with tumor antigens demonstrated safety and feasibility, though no significant T cell responses were observed. Subsequent analysis suggested the observed anti-tumor activity may stem from NK cell activation and enhanced tumor infiltration [111]. However, the safety and efficacy of DC-derived EVs as tumor vaccines require further study. More recently, a melanoma trial utilizing plasmacytoid DC (pDC)-derived EV vaccines administered via subcutaneous injection showed both safety and the capacity to induce robust T cell-mediated anti-tumor immunity [112]. These contrasting outcomes highlight the need for further investigation into the safety profiles and therapeutic efficacy of DC-EV vaccines.

Beyond DCs, TDEVs themselves represent promising platforms for vaccine development. In pancreatic ductal adenocarcinoma (PDAC), engineered EVs from immunogenic dying tumor cells carrying CCL22 siRNA effectively disrupt the CCR4/CCL22 axis between DCs and Tregs, thereby inhibiting Treg expansion and preventing tumor growth. In combination with first-line chemotherapeutic drugs, these modified EVs demonstrate synergistic anti-tumor effects [113]. Huang et al. developed an engineered TDEVs rich in α-lactalbumin and introduced immunogenic cell death (ICD) inducers and TLR3 agonists. This engineered EV can induce cancer cell ICD, thereby releasing tumor antigens and TLR3 agonists. It promotes the activation of type one conventional DCs and CD8 T cell-mediated anti-tumor responses, thereby inhibiting the growth of triple-negative breast cancer tumors [114].

#### 4.1.3 Drug Delivery

EVs have emerged as superior drug delivery vehicles due to their excellent biocompatibility, targeting properties, and cargo-protective capabilities [18]. Various therapeutic drugs can be loaded onto EVs *in vitro*, including small molecule drugs, nucleic acids, proteins, and nanoparticles [115,116]. EV-packaged small molecule chemotherapy drugs exhibit enhanced targeting efficiency, anti-tumor effects, and bioactivity [116].

As discussed above, functional RNAs exert important biological functions through intercellular crosstalk via EVs. Consequently, the therapeutic potential of loading therapeutic nucleic acid molecules, such as miRNAs, siRNAs, and CRISPR-Cas9, into EVs is increasingly being explored. MSC-derived EVs have emerged as effective miRNA delivery vehicles for miRNA-based therapies, demonstrating precise tissue targeting in various cancers, and significant anti-tumor effects across multiple cancer types [117,118]. While siRNA therapeutics offer precise gene silencing capability, their clinical translation has been limited by poor delivery efficiency, rapid degradation, and off-target effects. The membrane structure and targeting properties of EVs can facilitate the delivery of siRNAs to target organs [119]. The synergistic use of multiple anti-tumor molecules can significantly enhance therapeutic efficacy. Encapsulating both target gene-targeting siRNAs and anti-tumor drugs within EVs exhibits superior anti-tumor efficacy compared to monotherapy [120].

EVs functionalized with diverse metal nanoparticles represent a sophisticated, emerging platform for precision tumor therapy. Superparamagnetic iron oxide nanoparticles (SPIONs) enable magnetic field-guided tumor accumulation, significantly enhancing the delivery efficiency of EV-encapsulated chemotherapeutics [121]. Gold nanoparticles confer photothermal properties, converting absorbed light into localized hyperthermia for tumor ablation [122]. In combination with anti-miR-21, SPIONs and gold nanoparticles loaded simultaneously into EVs can achieve efficient photothermal therapy and anti-tumor effects [123].

EV-based therapeutic strategies hold significant potential, as evidenced by a growing body of clinical research. Although current trials have shown good safety profiles with infrequent serious adverse events, efficacy outcomes have been limited. Most studies do not reach their predefined efficacy endpoints, and the rate of patient benefit remains low (Table 1).

**Figure 3:**
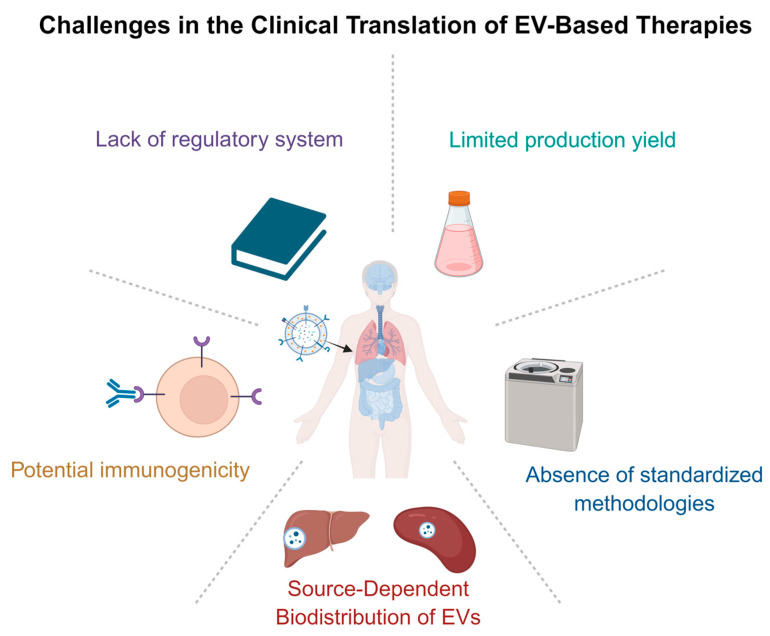
Challenges in the Clinical Translation of Extracellular vesicle (EV)-Based Therapies. The clinical translation of EV-based therapies is hindered by several challenges. Key obstacles encompass limited production yields that impede scalable manufacturing, the absence of standardized methodologies undermining batch-to-batch reproducibility, and considerable heterogeneity in the biodistribution of EVs derived from different sources, which complicates predictable *in vivo* delivery. Furthermore, potential immunogenicity raises safety concerns regarding adverse reactions, while the lack of a clear regulatory system presents a significant barrier to clinical development and approval.

Additionally, the translation of EV-based therapies faces several key challenges (Fig. 3). First, limited production yield hinders the scalability of EV-based treatments. Secondly, the absence of standardized methodologies for EV isolation and purification poses a major obstacle. The lack of a consensus protocol results in substantial batch-to-batch and inter-laboratory heterogeneity, thereby giving rise to poor reproducibility, inconsistent therapeutic efficacy, and a lack of robust quality controls, all of which hinder the scalable manufacturing and clinical translation of EV-based therapeutics. Third, endogenous and exogenously administered EVs differ significantly in their biodistribution. Systemically delivered exogenous EVs are often rapidly cleared or accumulate in organs like the liver and spleen, whereas endogenous EVs show different targeting preferences. Consequently, this mismatch may prevent designed EV therapeutics from reaching their target tissues effectively. Fourth, the immunogenic potential of EVs raises important safety considerations for clinical translation. Specifically, tumor-derived or allogeneic EVs may induce unintended immunomodulatory effects or, in some contexts, promote tumor progression. Finally, the translational path is further complicated by the current lack of rigorous regulatory frameworks, which fail to provide clear quality standards and application guidelines for EV-based therapeutics.

**Table 1:** Clinical trials involving EVs in cancer therapy.

	EVs Resources	Cargo	Disease	Outcomes	Phase	NCT Number/References
**Tumor vaccine**	DCs	Tumor antigen	NSCLC	Not yet published	Phase II trial	NCT01159288
	Autologous DCs	MHC/peptide complexes	Metastatic melanoma	No grade II toxicity; MR: 1; PR:1; SD:1; PD: 1*	Phase I trial	[111]
	Plasmacytoid dendritic cells	MAGE 3 peptides	III/IV melanoma	Tolerated; no serious vaccine-induced side effects; PR:1	Phase I trial	[112]
	DCs	IFN γ	NSCLC	Grade three hepatotoxicity; the median time to progression was 2.2 months and median overall survival was 15 months.	Phase II trial	[124]
	Autologous DCs	MAGE-A3, -A4, -A10, and MAGE-3DPO4 peptides	NSCLC	Tolerated with only grade 1–2 adverse events.	Phase I trial	[125]
**Drug delivery**	Plant	Curcumin	Colon Cancer	Not yet published	Phase I trial	NCT01294072
	Mesenchymal stromal cells	KrasG12D siRNA	Metastatic pancreas cancer with KrasG12D mutation	Tolerated with no treatment-related adverse events and increase in intratumoral CD8^+^ T cells.	Phase I trial	NCT03608631
	N.A.	STING agonist and PTGFRN	HNSCC, triple negative breast cancer, anaplastic thyroid carcinoma and cutaneous squamous cell carcinoma	Not yet published	Phase I/II trial	NCT04592484
	N.A.	STAT6 anti-sense oligonucleotide	HCC and patients with liver metastases from either primary gastric cancer or colorectal cancer	Not yet published	Phase I trial	NCT05375604

Note: MR: Minor response; PR: Partial response; SD: Stable disease; PD: Progressive disease. DCs: dendritic cells; NSCLC: non-small cell lung cancer; NCT: national clinical trial; MHC: major histocompatibility complex; MAGE: melanoma antigen gene; IFNγ: interferon-γ; CD8: cluster of differentiation 8; STING: stimulator of interferon genes protein; PTGFRN: prostaglandin F2 receptor inhibitorProstaglandin F2 receptor inhibitor; STAT6: signal transducer and activator of transcription 6; HCC: hepatocellular carcinoma.

### 4.2 Frontier Prospect of EVs

Despite significant progress, most EV research is constrained by non-specific isolation methods that overlook the inherent heterogeneity of EVs by evaluating only their ensemble functions. Recent advances in single-vesicle analysis technologies have enabled detailed characterization of the heterogeneity in the size and surface phenotype of individual vesicles [126]. Furthermore, EVs exhibit significant heterogeneity in protein expression, suggesting that distinct EV subpopulations may perform markedly different functions [127]. However, current understanding of EVs function within TME is primarily derived from studying the collective behavior of total EVs isolated from specific cell lines. Omics sequencing and characterization at the single-vesicle level are therefore crucial for functional analysis and clinical translation of specific EV subsets [128]. Mirroring the rapid development of single-cell sequencing technologies, methods for EV classification and single-vesicle analysis, such as Droplet Digital Analysis, Nanoparticle Tracking Analysis (NTA), Interference Correlation Microscopy and high-sensitivity Flow Cytometry have also seen substantial progress [129,130]. These single-vesicle analysis platforms are now being explored for clinical diagnostics. The high-throughput analysis of EVs from serum or plasma samples has demonstrated significant potential for early disease detection [131,132]. Employing a high-sensitivity flow cytometry-based single-vesicle analysis platform, CD147-positive EV subpopulations can be specifically identified. This capability holds significant promise for advancing EV-based liquid biopsy strategies in both early detection and prognostic evaluation [133]. Infrared and Raman spectroscopy have been utilized to detect specific molecular signatures of prostate cancer-derived EVs, showing their potential utility in prostate cancer screening [134].

Although holding great promise, single-vesicle analysis technology is hampered by considerable technical challenges that hinder its clinical translation. The foremost challenge is cost, with current methodologies relying on costly instrumentation that impedes both clinical accessibility and broad adoption. Thus, developing highly sensitive, rapid, and economical detection platforms, including user-friendly microfluidic chips and portable biosensors, represents a critical unmet need for enabling point-of-care testing. Third, standardization remains a pressing issue that must be addressed for the advancement of single-vesicle analysis technology. Furthermore, the biological mechanisms underlying the heterogeneity of EVs revealed by single-vesicle analysis remain incompletely understood, warranting more focused research. Additionally, clinical studies validating its utility in early detection, diagnosis, and prognosis prediction are still lacking; more translational research is therefore needed to identify robust EV-based biomarkers and drive clinical implementation.

The advancement of single-vesicle analysis platforms is also poised to foster the advancement of EV-based cancer therapeutics. This technology advances the field by providing a more comprehensive profiling of tumor-associated EV signatures, thereby enhancing the accuracy of early detection and prognostic assessment. Simultaneously, it enables the detection of low-abundance EV features, which paves the way for developing personalized treatment strategies and targeted therapies.

## 5 Conclusions

EVs mediate complex intercellular crosstalk within TME through their diverse molecular cargo, including proteins, nucleic acids, and metabolites. These EV-transported biomolecules exhibit cargo-dependent functions, capable of both promoting and suppressing tumor progression. Furthermore, EVs carry tumor-specific information, making them promising diagnostic and translational biomarkers. A growing number of research is elucidating the biological mechanisms by which EVs promote tumor progression, providing a theoretical basis for the development of future therapeutic strategies.

EVs have emerged as transformative platforms for cancer therapy due to their biocompatibility, natural targeting capabilities, and remarkable stability in circulation. The combination of immune checkpoint blockers, tumor vaccines, anti-tumor drugs, and nucleic acid molecules with EVs has promising anti-tumor effects. Multifunctional, engineered EVs can even demonstrate superior therapeutic efficacy compared to single agents. Numerous clinical trials have been conducted. Nevertheless, the translation of EV-based therapeutics into clinical practice still confronts several challenges. These include the need for standardized manufacturing and quality control protocols, more efficient and cost-effective technologies, and further in-depth investigation. As these issues are addressed in the future, EV-based therapies are poised to demonstrate significant potential.

## Data Availability

Not applicable.
